# Limited evidence of pseudoprogression following immune checkpoint inhibitor (ICI) therapy in glioblastoma

**DOI:** 10.1093/noajnl/vdaf232

**Published:** 2025-10-24

**Authors:** John Y Rhee, Juan Pablo Ospina Botero, Thomas Nelson, Kun Wei Song, Michael W Parsons, Elizabeth R Gerstner, Jorg Dietrich

**Affiliations:** Center for Neuro-Oncology, Department of Medical Oncology, Dana-Farber Cancer Institute, Harvard Medical School, Boston; Division of Adult Palliative Care, Department of Supportive Oncology, Dana-Farber Cancer Institute, Harvard Medical School, Boston; Center for Neuro-Oncology, Department of Medical Oncology, Dana-Farber Cancer Institute, Harvard Medical School, Boston; Department of Neurology, Division of Neuro-Oncology, Massachusetts General Hospital, Harvard Medical School, Boston; Division of Neuro-Oncology, Department of Neurosurgery, University of California San Francisco, San Francisco; Department of Neurology, Stanford University Medical Center, Palo Alto; Department of Neurology, Division of Neuro-Oncology, Massachusetts General Hospital, Harvard Medical School, Boston; Department of Neurology, Division of Neuro-Oncology, Massachusetts General Hospital, Harvard Medical School, Boston; Department of Neurology, Division of Neuro-Oncology, Massachusetts General Hospital, Harvard Medical School, Boston

**Keywords:** glioblastoma, immune checkpoint inhibitor, pseudoprogression

## Abstract

“Pseudoprogression” following immune checkpoint inhibitors (ICI) in glioblastoma is often considered in case of radiographic progression. To better characterize the frequency of this phenomenon in glioblastoma, we reviewed the imaging response characteristics of a total of 55 patients treated with ICI in the setting of recurrent (*n* = 45) or newly diagnosed (*n* = 10) disease. There was no evidence of pseudoprogression related to ICI-monotherapy in the entire cohort.

## Abstract

Glioblastoma is a high-grade malignant brain tumor. Even with standard-of-care treatment with surgical resection, concurrent chemoradiotherapy, and adjuvant chemotherapy, more than 90% of patients will experience disease progression, with median overall survival (OS) of 16-18 months and five-year survival rates at less than 10%. At recurrence, mean overall survival ranges from 6-11 months due to a lack of effective treatments.^1^

Immunotherapy with immune checkpoint inhibitor (ICI) blockade has been a powerful treatment option in several solid cancers, but positive results have so far not been replicated in glioblastoma. A phase 3 clinical trial demonstrated that nivolumab (PD-1 blockade) did not improve survival in patients with recurrent glioblastoma compared with bevacizumab.^2^ Two phase I trials similarly showed low progression free survival rates. Checkmate-143 had median progression free survival of 1.9, 1.5, and 2.1 months for nivolumab, nivolumab + higher dose ipilimumab,^3^ and higher dose nivolumab + ipilimumab, and atezolizumab showed a median survival of 4.2 months.^4^ However, ICI in combination with surgical treatment suggested a possible improved survival.^5^ Though this improvement in overall survival was not confirmed in a stage 2 single-arm expansion cohort, the study evaluated the cell cycle gene signature associated with neoadjuvant pembrolizumab and performed bulk-RNA seq on resected tumor tissue which demonstrated pharmacodynamic effect of anti-PD1 therapy in glioblastoma,^6^ and while this study was not sufficiently powered to address this question, this is currently being further investigated in a prospective study using dual ICI treatment with nivolumab +/− ipilimumab (NCT04606316).

The possibility of pseudoprogression following immunotherapy has been observed in systemic cancer but is poorly characterized in glioblastoma. Studies have shown pseudoprogression with ICI use in solid tumors, occurring in as high as 10% of patients, depending on tumor type and criteria used.^7^ In fact, iRANO in neuro-oncology,^8^ and iRECIST^9^ in systemic oncology were developed, in part, to account for pseudoprogression in clinical trials. The concern for pseudoprogression in glioblastoma treated with ICI is often raised in the clinical setting when trying to determine whether increased contrast enhancement on interval imaging may represent true progression or pseudoprogression.

In the current study, we aimed to characterize the frequency of pseudoprogression in a cohort of 55 patients with glioblastoma treated at Massachusetts General Hospital between 2015 and 2022 with ICI-based therapy, either in the newly diagnosed or recurrent setting. Treatment included pembrolizumab (29, 52.7%), durvalumab (19, 34.5%), nivolumab (6, 10.9%), or a combination of nivolumab and ipilimumab (1, 1.8%). Patients were identified from an institutional database that includes patients treated on clinical trials. Best radiographic and clinical responses after initiation of ICI were assessed in both groups following the Immunotherapy Response Assessment in Neuro-Oncology (iRANO) criteria by a neuro-oncologist (JR) and confirmed by two other investigators (JO, TN).^8^ Pseudoprogression was defined as new or increased contrast enhancement within the prior radiation field occurring within six months after receiving ICI, consistent with iRANO criteria.^8^ Tumors possessing isocitrate dehydrogenase mutations were excluded from this analysis. Molecular profiling data, including mismatch repair (MMR) and EGFR status, were abstracted from the electronic medical record when available. Testing was performed using a targeted next-generation sequencing panel (OncoPanel, Dana-Farber Cancer Institute) or, in some cases, by immunohistochemistry according to institutional protocols. O6-methylguanine-DNA methyltransferase (MGMT) promoter methylation data was also collected due to its relevance for prognosis in glioblastoma^10^ as well as rates of pseudoprogression.^11^

We identified a total of fifty-five patients treated with ICI for newly diagnosed (*n* = 10) or recurrent disease (*n* = 45) (Table 1). In the *newly diagnosed* cohort, the average age was 57 years. Molecular characteristics revealed the presence of MGMT promoter methylation in 10% (1/10) and EGFR amplification in 50% (5/10). Mismatch repair (MMR) was absent in this cohort. The median number of ICI cycles received was 9 (range 5-75). Most patients (8/10; 80%) received concurrent radiation therapy. Average dexamethasone prior to ICI initiation was 0.64 mg per day. Average dexamethasone use during ICI therapy was 1.60 mg per day. Median progression-free survival was 8.4 months (range 1.8-70.3 months), with the best ­radiographic response identified as stable disease (SD) in 90% at 2 months. No patient discontinued ICI due to toxicity. Only one patient from the newly diagnosed group was identified with evidence of “pseudoprogression” by Response ­Assessment in Neuro-Oncology (RANO) 2.0 criteria^12^—noted within two months after completion of radiation therapy (Figure 1).

**Table 1.** Characteristics of patients with glioblastoma (GBM) treated with immune checkpoint inhibitor (ICI)

	Newly Diagnosed GBM (*n* = 10)	Recurrent GBM (*n* = 45)
Age at diagnosis, mean (range)	57 years	57 years
	(45-71)	(29-78)
Gender, female (*n*, %)	2 (20)	15 (33)
MGMT methylation-status (*n*, %)		
Methylated	1 (10)	16 (36)
Unmethylated	9 (90)	28 (62)
Missing	0 (0)	1 (2)
Mismatch repair (*n*, %)		
Proficient	7 (70)	10 (22)
Deficient	0 (0)	33 (73)
Missing	3 (30)	2 (4)
Tumor laterality (*n*, %)		
Right	6 (60)	17 (38)
Left	4 (40)	27 (60)
Bilateral	0 (0)	1 (2)
EGFR-amplification (*n*, %)	5 (50)	20 (44)
Immune checkpoint inhibitor (ICI) received (*n*, %)		
Nivolumab	2 (20)	4 (9)
Pembrolizumab	0 (0)	29 (64)
Durvalumab	8 (80)	11 (24)
Nivolumab + Ipilimumab	0 (0)	1 (2)
Median ICI cycles received (range)	9 (5-75)	4 (1-37)
Concurrent bevacizumab with ICI (*n*, %)	0 (0)	21 (47)
Concurrent radiation (*n*, %)	8 (80)	8 (18)
Median progression free survival in months (range)	8.4 (1.8-70.3)	2.5 (0.4-35)
Best radiographic response at 2 months (*n*, %)		
Complete response	0 (0)	0 (0)
Partial response (PR)	0 (0)	4 (9)
Stable disease (SD)	9 (90)	21 (47)
Progressive disease (PD)	1 (10)	20 (44)
Pseudoprogression (PP) (*n*, %)	1 (10)	0 (0)

Abbreviation: MGMT, O6-methylguanine-DNA methyltransferase.

**Figure 1.**
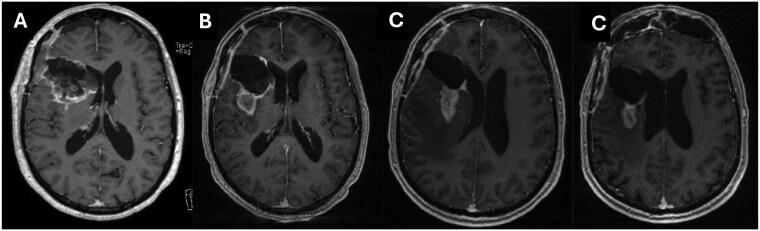
Brain MRI findings before and after receiving immune checkpoint inhibitor therapy. (A). Axial T1-post contrast MRI from a patient with newly diagnosed glioblastoma post-surgery and prior to initiation of 6-weeks of radiation therapy combined with durvalumab. (B). Axial T1-post contrast MRI 2 months after completion of combined radiation and ICI therapy, showing increase in nodular contrast enhancement. (C). Further increase in nodular enhancement at 2.5 months post radiation. (D). Decrease in nodular enhancement at 4 months post radiation, consistent with pseudoprogression.

In the cohort of glioblastoma patients treated with ICI at time of recurrence (*n* = 45), about half (*n *= 21; 47%) received concurrent bevacizumab, and some patients also received concurrent radiation therapy (8; 18%). The average age in this cohort was 57 years. Molecular characteristics revealed the presence of MGMT promoter methylation in 36% (16/45), EGFR amplification in 44% (20/45) and MMR deficiency in 73% (33/45).

The most frequently used ICI was pembrolizumab (29; 64%), followed by durvalumab (11; 24%). Grade 2 or higher ICI-related toxicity (headache, aphasia, granulomatous folliculitis, and acneiform rash) was identified in 9% (4/45), for which ICI therapy was temporarily held.

The median number of ICI cycles administered was 4 (range 1-37), and the median progression-free survival was 2.5 months (range 0.4-35 months). Best radiographic response in recurrent glioblastoma was PR in 9% (4/45), but all patients with PR received concurrent bevacizumab. This reflects results from the Avastin in Glioblastoma (AVAglio) study, where bevacizumab was shown to reduce the risk of pseudoprogression without improving overall survival.^13^ SD was seen in 47% (21/45) at 2 months. None of the patients treated at recurrence with ICI-based therapy had radiographic evidence of pseudoprogression. Of patients receiving ICI alone at recurrence, median overall survival was 4.8 months (*n* = 5 patients).

Collectively, we identified only one patient with evidence of radiographic pseudoprogression in the entire cohort of 55 patients, which was attributed to prior radiation therapy in newly diagnosed glioblastoma where this phenomenon is classically encountered within 3 months post irradiation.^14^ We did not encounter radiographic pseudoprogression attributed to ICI therapy. Of note, the presence of MMR deficiency or MGMT status did not correlate with treatment response and did not influence the occurrence of pseudoprogression following ICI therapy.

Though there have been reported transient increases in T2/FLAIR and enhancing lesions on MRI after dendritic cell vaccination^15^ and chimeric antigen receptor T-cell trials in glioblastoma,^16^ pseudoprogression is not reflected in other ICI-based trials (e.g. Checkmate-143)^3^ and was also not present in our cohort. The limited efficacy of ICI in treating glioblastoma may be related to the lack of a robust T-cell response.^14^ Further research has shown that in glioblastoma, even after PD-1 checkpoint blockade, macrophages and monocytes still constitute the majority of infiltrating immune cells, and sustained high expression of T-cell-suppressive checkpoints in these myeloid cells may prevent activation of the tumor infiltrating T cells.^17^ This may also explain the lack of pseudoprogression, ie lack of a positive inflammatory response, following ICI therapy in this disease. Urban et al examined ICI-related pseudoprogression in a mixed cohort of brain cancer patients and only identified one glioblastoma patient (∼0.8%) out of 123 patients treated with ICIs for various brain malignancies (mostly metastatic disease) developed pseudoprogression after ICI; however, no details or imaging characteristics were reported for this patient.^18^ In the Checkmate-143 study, pseudoprogression with nivolumab was also not reported.^3^ While pseudoprogression may occur in up to ∼40% of patients with glioblastoma treated with combined temozolomide and radiation therapy,^19^ the incidence in other solid tumors is generally < 15%.^20^

In conclusion, the data from our institutional cohort suggests that there is limited evidence of pseudoprogression in glioblastoma treated with ICI therapy and that increased contrast enhancement on MRI following ICI therapy in glioblastoma may be driven by true disease progression. Limitations to our study include the single-center study and the overall relatively small sample size addressing this issue of pseudoprogression after ICI therapy in glioblastoma.

## Data Availability

Data can be made available upon request to the corresponding authors.
